# Analysis of the cognitive processes involved in creating and sustaining cooperative group activity

**DOI:** 10.3389/fpsyg.2022.1038309

**Published:** 2022-12-07

**Authors:** Simon Skau

**Affiliations:** ^1^Institute of Neuroscience and Physiology, University of Gothenburg, Sahlgrenska Academy, Gothenburg, Sweden; ^2^Department of Pedagogical, Curricular and Professional Studies, Faculty of Education, University of Gothenburg, Gothenburg, Sweden

**Keywords:** shared intentionality, shared intention, group agency, cooperative activity, collective intentionally, cognitive change, cognitive development

## Abstract

A cooperative group activity (CGA) and shared intentionality are two phenomena whereby two or more individuals engage in an activity with the intention that the group will succeed, that is, to act as a “we. ” This ability to act together as a “we” is an important human psychological feature and has been argued to demarcate an important developmental step. Many CGA and shared intentionality theories have centered around philosophical problems of what counts as a “we” and how to give a cognitively plausible account of children's engagement in such activities, e.g., pretend play by toddlers. The aims of this paper are (i) to highlight the importance of distinguishing between creating and sustaining a CGA, since they require different cognitive abilities, (ii) to give a cognitively plausible account of the creation of a CGA, and iii) to present a formal framework of the sustainability of a CGA that can illuminate how engagement in a CGA stimulates cognitive change in its members. In the first part (section Creating cooperative group activity) of the paper, several theoretical problems are discussed, including the *common knowledge problem, the jointness problem, the central problem*, and the *cognitively plausible explanation problem*. The section ends with a cognitively plausible account of the creation of a CGA. The second part (section Sustainability of cooperative group activity) of the paper presents a formal framework of belief compatibility and trust relations. It explores how engagement in a CGA places certain cognitive constraints on its members while stimulating cognitive change and development. The paper ends with a discussion of empirical postulations derived from this account.

## Introduction

The ability to act with and in a group is an important human feature. We can distinguish between *cooperative* and *coordinated* group activities (Rakoczy, 2006). A cooperative group activity (CGA) refers to the phenomenon of two or more individuals engaging in a task with the intention that the group will succeed, which Tuomela calls we-mode we-intention (Tuomela, 2003), constitutive of pretend play (Rakoczy, 2006). On the contrary, a coordinated group activity refers to the phenomena where two or more individuals act in relation to each other with the main purpose of making a successful individual action by engaging in the group activity, which Tuomela calls I-mode we-intention (Tuomela, 2003), where driving on the “right side” of the road is a paradigmatic example. The current paper will focus on an analysis of a CGA.

It has been argued that collective intentionality is a prerequisite of a CGA (Bratman, 1999; Rakoczy, 2006). Coined by Searle in 1990, collective intentionality is intuitively understood as the phenomenon of two or more individuals acting as a group (Searle, 1990). Today, the phenomenon of collective intentionality is recognized as two vital and categorically different phenomena, namely, *shared* and *collective* intentionality (Tomasello and Rakoczy, 2003; Tomasello et al., 2005; Carpenter and Liebal, 2011). Shared intentionality refers to something that an agent is involved in, together with one or a few other agents, when the agent has an internalized common perspective with these specific agents in a specific context. Shared intentionality is what children are capable of doing after 1 year of age. Collective intentionality means an agent has an internalized perspective with a general agent, which is characterized by a more abstract set of perspectives and norms (Tomasello and Rakoczy, 2003). This means that the ability to perspective shift needs to be on such a developed level that the child can perceive others as mental agents with beliefs that differ from their own or are false. Tomasello et al. (2005) have argued that the ability of shared intentionality is acquired after the 9-month revolution and is a cognitively unique ability that separates us from other primates [but see (Moll et al., 2021) for a review and argument for a more gradual acquiring of shared intentionality]. Not only is shared intentionality uniquely human, they argue, but it is also argued that collective intentionality is just a development of shared intentionality (Rakoczy and Tomasello, 2007). The argument for the uniqueness is based on experimental studies where researchers interact in social problem-solving games with either toddlers/children or chimpanzees. In these studies, when the researcher stops their activity, the children (18–24 months old), but not the chimpanzees, attempt to reengage the researcher in the shared activity, which is interpreted as an interest in and recognition of the joint commitment for the children, but not the chimpanzees (Warneken et al., 2006), and children (34–40 months old), not chimpanzees, continue to collaborate even if they could get the reward by themselves (Rekers et al., 2011). In contrast to this view, several observational studies of interactions have provided evidence of nonhuman primates (including chimpanzees) engaging in shared intentionality during imitation games (Persson et al., 2018) and rough-and-tumble play (Heesen et al., 2017), satisfying the criteria of cooperation ascribed to toddlers, for example, being motivated to collaborate for noninstrumental reasons, giving credence to the theory that the evolutionary roots of shared intentionality are shared with at least other great ape species (Persson et al., 2018).

Although intentionality and intention are not the same thing, where the former is a property of a mental state to be about an object or state of affairs and the latter is one type of mental state that has the property of intentionality. I will follow the tradition utilized in recent years in psychology by using it in a more specific sense. In this more specific sense, intentionality is a property of an intentional action, such as intentions; thus, shared intentionality will refer to “the capacity to share conative attitudes and, more specifically, intentions” (Salice and Henriksen, 2021, p. 3). Thus, this analysis of shared intentionality will be an analysis of shared intentions.

Much research has been about what cognitive requirement makes shared intention and a CGA possible. This paper will highlight the difference between what is needed to create a CGA and what is needed to sustain a CGA. A framework will be presented that can explain and explore how a CGA stimulates cognitive change by focusing on the mechanism that sustains a CGA. In the first part, some of the questions and problems that need to be tackled in a theory of a CGA are presented and the section ends with a suggestion of a theory of shared intentionality. The second part explores some of the properties of the continuation of a CGA by formulating a formal model that explains how a CGA can stimulate cognitive change. The paper ends with a discussion of empirical postulations derived from this account.

## Creating cooperative group activity

### Framing the traditional problems

Traditionally, theorizing about shared intentionality can be understood as being centered around five questions. First, the *common knowledge problem*, how can any proposition X be “open” so that all individuals in a group know that everybody knows X? Second, the *jointness problem*, how can an experience be joint or shared (Carpenter and Liebal, 2011)? Third, the *central problem*, is how should we explain how two or more individuals engage in shared activities? Schweikard and Schmid summarized the central problem as the contradiction between the statements “(a) Collective intentionality is no simple summation, aggregate, or distributive pattern of individual intentionality (the Irreducibility Claim) [and] (b) Collective intentionality is had by the participating individuals, and all the intentionality an individual has is his or her own (the Individual Ownership Claim)” (Schweikard and Hans, 2013, p. 1). Traditionally, in spelling out how a group of individuals can be in a state that constitutes shared intention, theorists talk about collective actions and the intentions that bring them about. Fourth, how should we understand the normativity that stems from group action and shared activities? The first three questions are often dealt with at the same time, for example, philosophical theories of collective intentionality such as Bratman (1999), Gilbert (1989), and Tuomela (1995), use common knowledge, that is, to make something open, as a necessary criterion to make individual's intentions joint in a way that constitutes collective action.

The fifth question, originating from developmental psychology, is how we should give a cognitively plausible explanation for the fact that one-year-olds seem to be able to engage in all four of the phenomena mentioned above. A sixth question that I will not touch upon in this paper is whether shared intentionality and a CGA are a uniquely human ability or not. On physical cognition (space, causality, and quantities), humans perform comparably with chimpanzees and bonobos in the first years of their life. After 4 years of age, children become more advanced, whereas apes stay on their 2-year-old performance level. However, humans are already significantly more evolved at 2 years of age in social cognition (communication, social learning, and theory of mind), compared with chimpanzees and bonobos (Wobber et al., 2014). It has been argued that what separates us, seems to be that we and not our closest primates, have the motivation to engage in a CGA (Tomasello, 2020; O'Madagain and Tomasello, 2022). In reviewing the literature on the great apes, Call states: “It appears that chimpanzees, unlike humans, are not motivated to share interesting sights or work together when there is no need to do so” (Call, 2009, p. 373). For more information, see Tomasello et al. (2005), Call (2009), and Carpenter and Call (2013). For a contradictory view, see Boesch (2005), Heesen et al. (2017), Kaufmann (2020), and Persson et al. (2018).

Part of the aim of this paper is to highlight an additional problem that we need an account of a CGA that separates between the creation of a CGA and the sustaining of a CGA.

### The common knowledge problem

To illustrate how a fact can be open, suppose that every employer knows that the boss will be late, but nobody knows that everybody else knows this. This state of affairs is usually referred to as *mutual knowledge* (Vanderschraaf and Giacomo, 2014). However, when someone announces that they have spoken to the boss who says they will be late, the state of affairs changes from mutual knowledge to *common knowledge*. The fact that the boss is late has now changed to be *open* or *public*. So, if there is mutual knowledge in a group, then we must add *something* for it to be open. Traditionally, the problem is how we should articulate common knowledge. From a developmental psychology view, this articulation has been rather unsatisfactory. Common knowledge theories often presuppose an infinite iteration of “I know that you know … that I know” (Lewis, 1969; Schiffer, 1972).

For this reason, many have argued that any definition of common knowledge will be too strong because at a minimum it requires a fully developed mind with functioning mental faculties (Tollefsen, 2005; Rakoczy, 2006). Tollefsen (2005) argues that a child between the ages of 1 year and 4 years who has not yet developed a robust *theory of mind* can still be said to engage in situations where they use something being open in a group. Carpenter argues, contra Tollefsen, that there is evidence suggesting that even 1-year-old infants possess a robust theory of mind with the understanding of other goals, beliefs, and intentions (Carpenter, 2009). However, she states that this does not mean they fulfill the criteria of common knowledge, as it is traditionally defined.

Instead of requiring infinite iterations of “I know that you know … that I know' as Bratman's (1999), Gilbert's (1989), and Tuomela's (2005) theories require, Tomasello (2008) argues that the phenomena he calls *common ground* or *recursive mindreading* should be understood as *indefinite* to the effect that we only need to compute, as far as we need and can, which often only are a couple of levels up. Wilby argues that this truncated approach misunderstands the problem (Wilby, 2010). He reasons that the problem is not a mismatch between the psychological limitations of individuals and an idealized notion of what common knowledge should consist of. Rather, it is a mismatch between the psychological limitations of individuals and the need for a psychologically expedient notion of what common knowledge is^1^.

### Jointness as openness

Already in the first year of life, infants can evaluate actions and intentions. Eight-month-old, but not 5-month-old, infants can evaluate helping intentions (Hamlin, 2013) and 9–10-month-old infants can evaluate distribution intentions of actions regardless of outcome (Strid and Meristo, 2020; Geraci et al., 2022). A great deal of research shows that infants have some understanding of what is open to them and what is not because of what they have jointly experienced. Liszkowski et al. (2008) showed that 12-month-old infants point to a fallen object that an adult has not seen fall, compared with one, which they did see fall. Liebal et al. (2011) showed that an infant, who had played with two different toys with two different researchers, later entered a room with one of the researchers; the child pointed to a picture of the toy with which the child and researcher had a shared experience. Moll et al. (2008) constructed a study where 14-month-olds played with three toys and one adult. The adult showed excitement for one of the toys, and later, when the adult asked for a toy, they were given that one. In the control situation, a new adult came in and asked for a toy. The children were not more inclined to give them the special toy, rather just one at random. It is improbable that this understanding of what is open between the children and researchers amounts to common knowledge, as defined above. However, as Carpenter points out, “it is neither joint attention since these cases are not bound to perception either” (Carpenter, 2009, p. 383).

To explain cases like the toddlers in Moll et al. (2008), where there is openness but not common knowledge, some researchers have turned to the less demanding phenomena of joint attention. The debate on joint attention concerns both the openness and the jointness problem. Campbell differentiates between *reductive* and *relational* accounts of joint attention (Campbell, 2002, 2005). According to the reductive account, we should be able to express each participant's state without implying that there is joint attention. However, according to the relational view, joint attention is a primitive phenomenon of consciousness. Campbell argues a relational theory where the co-attender in the joint attention can figure as a constitutive of the attention experience without being the object of the attention. He claims this is a more accurate explanation of the easiness and simplicity of joint attention. Still, there is a problem with a relational account of joint attention or Wilby's theory of common knowledge (Wilby, 2010); it seems to smuggle in what it tries to explain. As Peacocke states: “If ‘co-attender' means something stronger and implies joint attention to the object to which both are attending, the notion of a co-attender simply embeds the property which is to be explained, the openness of joint attention” (Peacocke, 2005, p. 299).

Peacocke, on the contrary, presents a reductive theory of joint attention based on perception and what he calls *mutual open-ended perceptual availability*:

“Each perceives that the other perceives that s obtains; and if either is occurrently aware that the other is aware that he is aware… that s obtains, then the state of affairs of this being so occurrently aware is available to the other's occurrent awareness” (Peacocke, 2005, p. 302).

This account may capture *something* that causes children to engage in openness. Nevertheless, it can only be a sufficient condition, as it is based on our perceptual faculties for how children can engage in openness and not a necessary condition. If we take 14-month-olds in Moll et al. (2008), they seem to know what information was open where there was no obvious perceptual answer. Rather, they knew from the context and their previous experience which toy the adult wanted. One can appeal to memory, but that would be to sub-optimize and would not solve more complex openness problems, such as coordination attacks (Wilby, 2010). Neither Campbell nor Peacocke answers the openness and the jointness problems satisfactorily.

### Communication, seem-openness, and joint attentiveness

Carpenter and Liebal (2011) sketch out a new theory that is not reductive or relational. They claim that sharing a joint attention and psychological state always involve communication, that is, we will at best have parallel attention without communication. They claim that a person sitting in a movie theater not interacting with anyone, should only be said to have parallel attention to the movie and not joint attention. What is needed is at least that “at some point you and the stranger [sitting next to you] turned to look at each other to smile about something one of the actors said or to remark on how good the movie was or the like” (Carpenter and Liebal, 2011, p. 167). Communication is not restricted to verbal or linguistic communication. An obvious example is children's declarative pointing, as mentioned above. However, they also claim that special looks, so-called communicative looks, like those infants give in face-to-face interaction, could be seen as communicative to the extent of joint attention.

Carpenter and Liebal do not think of this as a complete theory of joint attention, even though they endorse that communication is both necessary and sufficient for joint attention. However, communication does not necessitate that a fact will be open, for example, in any coordination attack. As illustrated in Campbell (2002), Campbell (2005), and Wilby (2010), we have successful communication, and every message they send can be interpreted into one of the communicative looks, without there being any openness between them.

There is a way in which communication can give us openness. The rationale behind communication as being necessary for joint attention is that it makes something public or open, which is the openness question. More specifically, something is public or open, if and only if it seems to be open. One might argue that it must first be intended to be open; however, there are many cases where a person feels something, for example, anger, but is not aware of it before it is pointed out that they are acting angrily. Here, we have our answer to the openness problem, that is, *something* that needs to be added in order for a group of individuals who possess mutual knowledge is that it seems open. If everyone knows X (the boss will be late) and it seems to everyone that everyone else knows X (the boss will be late), then X (the boss will be late) is open, thus solving the common knowledge problem. From this, we get what we can call *seem-openness*:

Bruce is *seem*-open to Arthur about X if and only if it looks/seems to Arthur as if Bruce is open about his intention regarding X.

“Open about one's intention” means giving a public indication about something, for example, if Arthur would like to go for a walk with Bruce, Arthur could tell Bruce that he wants to take a walk, which would mean that Arthur has made his intention open for Bruce. As we saw in the previous section, infants can evaluate actions and intentions (Hamlin, 2013; Strid and Meristo, 2020; Geraci et al., 2022). Tomasello and Rakoczy (2003) discuss the phenomenon of *tuning in*. When children reach 9 months of age, they begin to have triadic perceptions. However, some studies, for example, Striano and Bertin (2005) and Grossmann and Johnson (2010), indicate that this ability of triadic perception is developed even in 5- and 7-month-olds. From this moment on, the child can *tune in* to others' behavior or perception toward objects or get other people to *tune in* to their behavior or interest at that moment (Tomasello and Rakoczy, 2003). With seem-openness, we can capture that phenomenon.

I will now present what I call *joint attentiveness*. I do not call it joint attention since it is not necessarily perceptual as Carpenter (2009) points out was not necessary. On the contrary, joint attentiveness will help to explain the shared intention and its jointness answers the openness problem. It is the case that Arthur and Bruce engage in joint attentiveness about X (e.g., seeing a bird) if and only if:

Bruce is aware that Arthur sees X and it seems to Arthur as if Bruce is open about being aware of Arthur seeing X, then Arthur believes that Arthur and Bruce are tuned in together. The same thing holds true for Bruce.

Here, we see that joint attentiveness is a constitutive state of affairs, to use Searlean terminology (Searle, 1983). Thus, we can say that Arthur and Bruce have joint attentiveness about X by-way-of Arthur and Bruce believing they are tuned in together. With this definition, we can describe young toddlers participating in openness and tuning in without subscribing to unrealistic cognitive processing. If we call the attention to something, the dyadic relation, for level 0, and the triadic awareness for level 1, then joint attentiveness requires the capacity to compute something on level 2. This is not more cognitively demanding than realizing that you are in a triadic perception.

### An account of cooperative group activity

Tollefsen (2005) also realizes the problem psychological research causes for philosophical theories of shared intentionality. To overcome this, she creates an account of a CGA compatible with children who do not possess a robust theory of mind. She reformulates Bratman's theory by changing the criterion of common knowledge with Peacocke's version of joint attention.

“1. (a) I intend that we J and (b) you intend that we J.
2. I intend that we J in accordance with and because of 1a and 1b, and
meshing subplans of 1a and 1b; you intend the same.
3. 1 and 2 are jointly perceived (or as Peacocke puts it, 1 and 2 are mutual
open-ended perceptually available)” (Tollefsen, 2005, p. 92–93).

The jointness of the shared intention is achieved by 1 and 2 being open between you and me. The openness is achieved by appealing to Peacocke's theory of joint attention, which we earlier saw did not explain all cases of openness, for example, 14-month-olds in Moll et al. (2008). However, the notion of joint attentiveness can, which is why a suggestion is that we change 3 to be: *there is joint attentiveness about 1 and 2*.

However, there is a problem with Bratman's notion of meshing subplans. Bratman's idea is that when two or more individuals intend to do something together in joint action, for example, paint a house, then the subplans of each agent must mesh. The subplans an individual has are the different strategies and sub-strategies that the individual wants to follow to reach the goal. For example, if Arthur has the subplan of using red paint and Bruce has the subplan of using blue paint, then one of them needs to change one of their subplans, or they will not participate in the joint action of painting together. With respect to the shared intention between Arthur and Bruce, meshing, in this case, means that there is some way they could jointly intend to do J without violating either of their subplans. For example, Arthur might want to use red paint but does not care about what type of paint, whereas Bruce does not care what color it is just as long as it is environmentally friendly paint. This is a case where Arthur and Bruce have meshing subplans. Important to note is that Arthur and Bruce do not need to know (every one of) the subplans of the other to have a joint intention. However, Bratman (1999) views the meshing subplans condition as part of the content of the intention that each agent has. He argues that if we do not have the meshing of our subplans in the content of our intention, then we do not ensure the commitment to fulfill the intention. However, this is unnecessary since the meshing of subplans follows conceptually from the intention that we do J together. The standard interpretation of intention that I intend to do J implies that I believe J is possible and that I desire to achieve J (Searle, 1983). This implication means that when I intend that we do J, I also believe it is possible that we can achieve J together. From the fact that I believe that there is a possibility for us to achieve J together, it follows that I also believe that we can make our subplans mesh, because, without it, we could not achieve J together. Also, if we both intend for us to do J and this is seem-open to us, then we both believe we have the same goal to achieve J together and the desire to achieve it. From this, we are both bound by the rationality constraint of the action intention, which Tuomela clarifies with his schemas W1 and W2:

“(W1) (i) We will do X. Therefore: (ii) I will do my part of X. (W2) (i) We will do X. (ii) X cannot be performed by us unless we perform action Z (for instance, teach agent A, who is one of us, to do something required of him for X). Therefore, (iii) We will do Z.: (iv) Unless I perform Y we cannot perform Z. Therefore [because of (iii) and (iv)], (v) I will do Y (as my contribution to Z)” (Tuomela, 2005, p. 342).

If our subplans do not, in fact, mesh, e.g., I do not want the same paint as you, so I am not ready to do my part in achieving J, for example, buy a color that we both agree on. This means that if I intend for us to do J, then I am also ready to do my part to achieve J and I cannot do my part or agree on you doing your part without meshing subplans. Most of the time, our subplans are not set in stone but are constantly being updated depending on our actions and subplans to the effect that they stay in a meshing state since the shared intention will otherwise dissolve. From this, we see that condition 2 is redundant and is derivable from condition 1.

Thus, we can reformulate Bratman's account: (1) I intend that we J and you intend that we J and (2) there is joint attentiveness about (1). This formulation of a CGA handles the common knowledge problem, the jointness problem, the central problem, and the cognitively plausible explanation problem, as discussed in section Framing the traditional problems.

### Consequence of creating a cooperative group activity

Formulating the theory of the shared intention in the third person would be:

1a) Arthur has the intention to do X together with Bruce.
1b) Bruce has the intention to do X together with Arthur.
ii) Arthur and Bruce have joint attentiveness about (1a) and (1b).

If (1) and (2) are fulfilled, Arthur and Bruce have a shared intention. Only following Arthur, we can now state that:

(i) Arthur has the shared intention to do X together with Bruce.

The difference between (1a) and (i) is that they are two different mental states, where (1a) alone does not generate (i) since it is the addition of (2) that makes it possible. The difference can be exemplified by the fact that in (1a), Arthur believes that he and Bruce *can* share the goal of doing X (playing) together, but in (i), Arthur believes that he and Bruce *do* share the goal of doing X (playing) together.

That this is a new mental state has three consequences. The first is that the fulfillment of conditions (1) and (2) causes this new mental state (shared intention). Second, the content of this new mental state refers back to, but does not contain, the mental states and the states of affairs in conditions (1) and (2). It is essential for a mental state to be able to refer back but not contain the previous mental state. The phenomenon is common, for example, we have all been in a situation where we have figured out a complex logical problem that mobilized all of our cognitive capacities. We can later be in a situation where we think about that problem and use the thinking we did when figuring out the problem without re-engaging in the act of figuring it out. In this way, we can use the complexity of what we have previously done by making part of the content of the mental state refer back but not contain it.

Thirdly, Arthur and Bruce have gone from time t_1_, when the joint attentiveness was constituted to time t_2_, where they have a shared intention. That Arthur and Bruce are now at time t_2_, distinct from t_1_, does not mean that forms of (1) and (2) cannot emerge again at time t_2_ or time t_n+1_; what it means is that they do not have to emerge again in order for there to be a shared intention.

Continuing to analyze the shared intention, it follows from

i) that:
ii) Arthur believes that they will achieve X together,
and
iii) Arthur desires to achieve X together. Continuing, it follows from the rationality constraint of intentions that if Arthur has a shared intention to achieve X together with Bruce, then
iv) Arthur intends to do his part of/to realize X (in accordance with schema W1 and W2 mentioned in the previous section).
Furthermore,
v) Arthur intends to do his part of X if and only if there are meshing subplans between Arthur and Bruce to the effect that X.

Conditions (iv) and (v) establish the action intentions and states of affairs that follow and are required for the sustainability of a shared intention and CGA. As this description of the shared intention shows, Arthur and Bruce will have meshing subplans if they fulfill (1) and (2). If it turns out at another time t_n_ that their subplans do not mesh, then the shared intention dissolves. This means that at t_1_, they could have meshing subplans, but at t_2_ they may not, which means that at t_2_, when (i) is fulfilled, the shared intention ceases. Borrowing terms from Amie Thomasson, we could say that the shared intention derives its existence from (1) and (2) and that (i)–(v) maintain its contingency^2^. What this account of the shared intention gives us is an explanation of the intuitive idea that there is a difference between the creation of a CGA and the sustainability of a CGA. The important consequence of this is that the frequency of shared intention, and a CGA, in a population (e.g., infants, toddlers, children, teenagers, adults, and animals), depends on the cognitive ability to engage in the creation of a CGA and in the cognitive ability to sustain a CGA.

We have now gone through a cognitively plausible account of what is needed of an individual to engage in the creation of a CGA, and in the next section, we will explore what is needed to sustain a CGA. This exploration will also center on how engaging in a CGA stimulates cognitive change in individuals.

## Sustainability of cooperative group activity

In this section, we will expand on what (iv) and (v) mean for the sustainability of a CGA. A consequence of (iv), explained in the W1 and W2 schemas, is that there needs to be some form of trust between the members of a CGA of a shared intention. More specifically, Arthur and Bruce have a shared intention to do X (perform a duet) if Arthur trusts Bruce to do his part of X (sing his lyrics) and Bruce trusts Arthur to do his part of X (sing his lyrics). In studies with the minimal group paradigm (Dunham, 2018) where individuals are grouped together based on arbitrary characteristics, adults' motivation to engage in shared intentionality increases (McClung et al., 2017). Although 3–4-year-olds can engage in a CGA, they do not trust in-group members more than out-group members only based on minimal groups (MacDonald et al., 2013; Plötner et al., 2015), whereas 5-year-olds do (Plötner et al., 2015).

From (v), we see that the subplans need to mesh. Here, we will mainly use the concept of *plans* to denote different types of beliefs: *facts* (e.g., dogs can bark), *preferences* (e.g., ice cream is the best dessert), and *ideologies* (e.g., there is only one God), where ideology contains elements of both facts and preferences (Heiphetz et al., 2013). In a recent study, Roberts et al. showed that the tendency to prescribe what beliefs an individual should have based on a description of the *group belief* the individual is a member of, is dependent on age and belief type. They found that 4–6-year-olds cared more about the truthfulness of fact statements and not about what a group believed compared with 7–9-year-olds and adults, whereas 7–9-year-olds approved less than adults when an individual had a divergent ideology than the rest of the group. Regarding preference, all three age groups had a higher rate of disapproval when the preference was divergent from the group compared with when it was the same (Roberts et al., 2021).

According to consistency theory, when there is inconstancy between the propositions of two or more beliefs, it causes tensions that the individual seeks to resolve (Gawronski and Brannon, 2019). Propositions X and Y are inconsistent if not X follows from Y (e.g., where X is “all opinions are equally valid” and Y is “some opinions are better than others”). Even though individuals can be faulty in their detection of (in)consistency, when an inconsistency is detected, they create a model in which X and Y are consistent, or one of the beliefs is dropped (Gawronski and Brannon, 2019). The credibility of proposition X sometimes depends on the credibility of other propositions Y and Z and their logical structure. To illustrate this, Friedkin et al. showed the logical structure of Colin Powell's speech in February 2003 to the UN Security Council and how it changed the public perception in the USA about invading Iraq:

“Statement 1. Saddam Hussein has a stockpile of weapons of mass destruction. Statement 2. Saddam Hussein's weapons of mass destruction are real and present dangers to the region and to the world. Statement 3. A preemptive invasion of Iraq would be a just war. It was a logic structure in which high certainty of belief on statement 1 implies high certainty of belief on statements 2 and 3 […] In the immediate March-May aftermath of the invasion, polling indicated a surge to strong majority support of the preemptive invasion. With the failure to find any evidence of weapons of mass destruction in Iraq, polling indicated that a strong majority of the public believed that the Iraq War was based on incorrect assumptions” (Friedkin et al., 2016, p. 322–323).

That children are sensitive to the logical structure was shown in a recent paper by Schleihauf et al. (2022) where they found that 4-year-olds' reliability changed their belief about X (e.g., the reward is in the blue box) when the credibility of another belief changed.

### Meshing subplans as compatibility

We have many beliefs and plans, but all of them are not involved in the meshing of subplans that constitute a CGA. To describe this, we can characterize an individual as a set of plans and call any situation or context φ in which a shared intention X is possible. We can denote this set of subplans as α = {α_1_, α_2_ … α_n_}. These subplans are the only ones relevant to the shared intention X and thus the only subplans that need to mesh. Using *A* to stand for all Arthur's plans and beliefs, we can state this formally as *A* ∩(*X*|φ) = α, X ∈ *A*, that is, the intersection between all plans and beliefs of an individual (*A*) and the shared intention X given the context φ, is α. This means that both α and X are proper subset of *A*.

The meshing of subplans means that the subplans between Arthur and Bruce need to be compatible. Let us denote the meshing of two subplans between Arthur and Bruce as M(α_n_,β_m_) and the meshing of all relevant subplans between Arthur and Bruce as **MS**(α.β) and the compatibility between these subplans as graded between 0 and 1. We will use M to denote the compatibility between two individual subplans and **MS** as the compatibility between two sets of subplans. From this, we have **MS**(α,β) = M(α_1_,β_1_) × M(α_1_,β_2_) × ... × M(α_n_,β_m_). The compatibility between two subplans or sets of subplans is denoted as follows:

M(α_n_,β_m_) = 1 =_df_ The subplans α_n_, and β_m_ are fully compatible.
**MS**(α, β) = 1 =_df_ The set of subplans α and β are fully compatible.
M(α_n_,β_m_)= 0 =_df_ The subplans α_n_ and β_m_ are not compatible.
**MS**(α, β) = 0 =_df_ The set of subplans α and β are not compatible.
M(α_n_,β_m_) = x where 0 < x ≤ 1 =_df_ The subplans α_n_ and β_m_ are compatible.
**MS**(α,β) = x where 0 < x ≤ 1 =_df_ The set of subplans α and β are compatible.

To illustrate this, we can use the example of Arthur and Bruce painting the wall together. Arthur has the subplan α_n_ to paint the wall red, and Bruce has the subplan β_m_ to paint the wall red, then they have M(α_n_,β_m_) = 1, that is, their subplans mesh and are fully compatible. If α_n_ is to use environmentally friendly paint and β_m_ is to use green paint, then M(α_n_,β_m_) = 1, that is, their subplans mesh and are fully compatible since they both intend to do the painting together, and these subplans do not conflict with each other at any level. However, if α_n_ is to use blue paint and β_m_ is to use red paint, then M(α_n_,β_m_) = 0, that is, their subplans are not compatible and do not mesh. Let us say the shared intention is to paint the fence the first week of May. Also, presuppose that α_n_ is that Arthur only plans to paint the fence on a Saturday and β_m_ is that Bruce does not care what day of the week it is. What we get then is M(α_n_,β_m_) = 1/7= 0.14, that is, α_n_ and β_m_ are compatible to a degree of 0.14, no matter what the external circumstances are. The external circumstances could be that there is a snowstorm the whole first week of May, so Arthur and Bruce cannot leave their houses, or it could be the case that the snowstorm stops on Saturday. Both are possible scenarios; however, low the possibility might be, we do not incorporate them in our definition of compatibility^3^. This means that the compatibility value M(α_n_,β_m_) = 0.14 is only dependent on the plans and beliefs of Arthur and Bruce. Important to note here is that if Arthur changes his plan α_n_ to α_k_, which is that Arthur plans to paint either Saturday or Sunday, that will give us M(α_k_,β_m_) = 0.28. This means that the new meshing of subplans is more compatible, not that the first was incompatible.

This explication of meshing subplans also implies that the individual's plans and beliefs need to be self-compatible, to the effect stated by the consistency theory. Although, as van Kampen points out, “that consistency is a fundamental principle of all cognitive processing and is not limited to propositional processes only” (van Kampen, 2019, p. 49), we are not always aware of the inconsistency of our plans and beliefs (until they are activated at the same time), we can be faulty in our detection of inconsistency on such a high cognitive level as beliefs (in contrast to perceptual inconsistency), and many of our plans are in a meshing state and are constantly updating. Nevertheless, this framework proposes that (i) it is the cognitive capacity of having consistency among plans and beliefs in relation to the shared intention X that will influence one's ability to participate in a CGA and (ii) the compatibility of subplans of a CGA stimulates cognitive change in its members.

### Density as group complexity

The formulation of meshing subplans estimates the compatibility between plans and belief sets between two or more individuals in a CGA. Another important part is the trust relation between the individuals in a CGA. As a working example, there are four individuals, namely, Arthur, Bruce, Clark, and Diana, who have a CGA. Following the account presented in 2.6, all individuals believe they will do it together. However, they may be connected in this special way to do X as a group of four, but how connected is the group? The measurement will denote the *density* (**D**) of the group.

The connections between the individuals should be understood as a directed relation, that is, Arthur's relation to Bruce is directed to Bruce, and Bruce has a different directed relation to Arthur. This means that this section can be translated into a symmetrical directed graph. The directed relations (or edges) between two individuals (or nodes) should intuitively be understood as a *trust* relation, and the degree of trust (the weights of the edges) between two individuals is graded between 0 and 1. We will denote the directed trust relation **T**, and just like with meshing subplans, the degree of the trust between Arthur and Bruce is dependent on a situation φ where a shared intention X is possible. We can write it as A**T**B ∩(*X*|φ)= degree of trust and define it as follows:

A**T**B ∩(*X*|φ) = 1 =_df_ A trusts B completely, for the shared intention X given context φ.
A**T**B ∩(*X*|φ) = 0 =_df_ A does not trust B at all, for the shared intention X given context φ.
A**T**B ∩(*X*|φ) = x where 0 < x ≤ 1 =_df_ A trusts B, for the shared intention X given context φ.

The measurement of the density **D** is the same as in regular graph theory. However, the density interpretation in graph theory differs somewhat. In the graph theory, a dense graph is a graph in which the number of edges is close to the maximal number of edges. The opposite, a graph with only a few edges, is a sparse graph. The distinction between sparse and dense graphs is rather vague and depends on the context in graph theory. In contrast, I will only use the term density (**D**) and talk about more or less dense groups.

**Figure 1:**
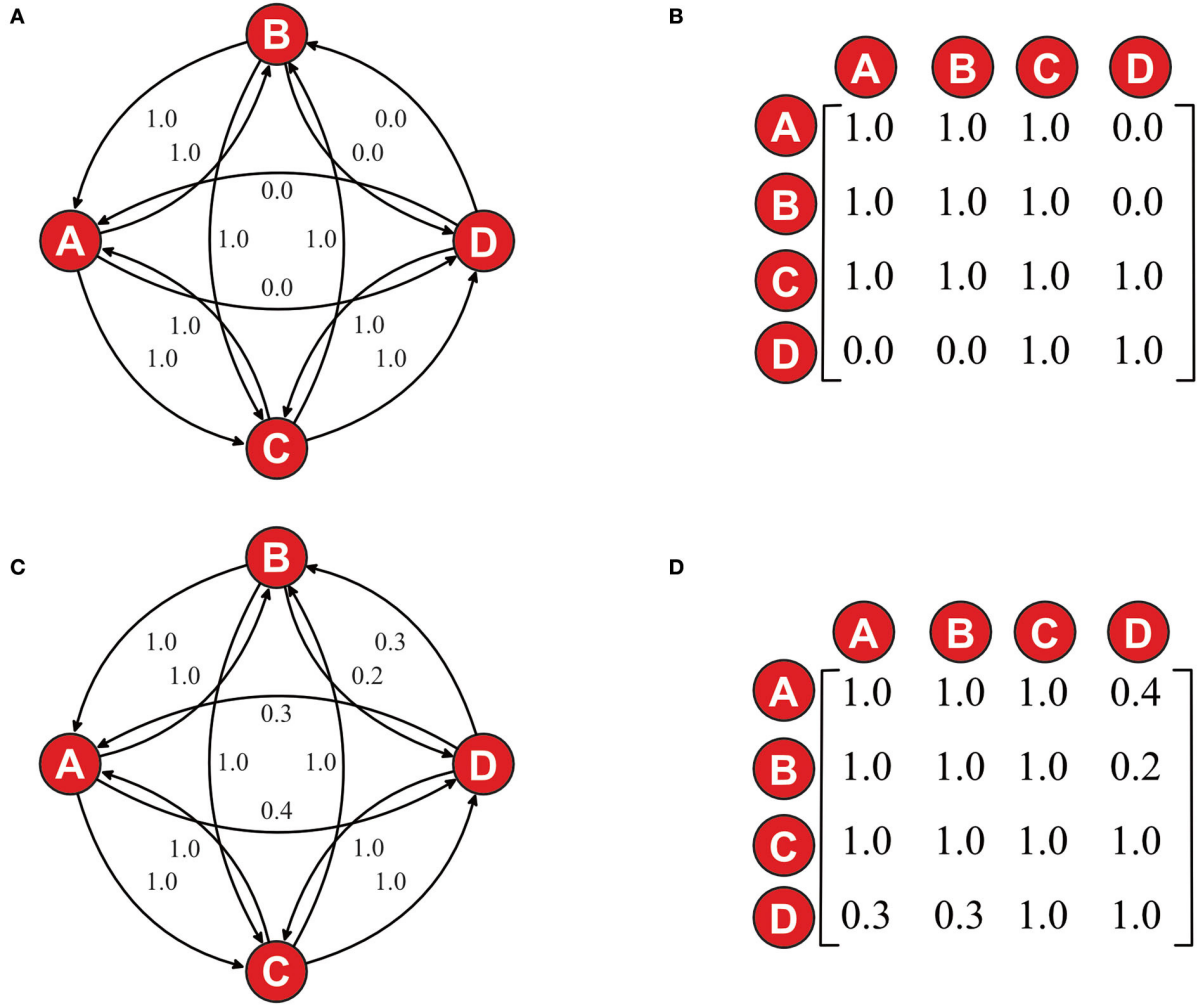
**(A)** Graphic representation of trust network. **(B)** Adjacency matrix of the same network as in **(A)**. **(C)** The same graphic representation of trust network as in **(A)** but with updated trust relations. **(D)** Adjacency matrix of the same network as in **(C)**. The diagonal A**T**A, B**T**B, C**T**C, and D**T**D are set to 1, even though there are no reflexive relation/arrows.

Let us say that Diana only participates in a CGA because Clark is participating. This would mean that given the shared intention X (doing a puzzle together), Diana is only connected to Arthur and Bruce in those cases and subgroups that Clark is involved in. Let us for simplicity's sake characterize this as D**T**A = 0, A**T**D = 0, D**T**B = 0, B**T**D = 0, and 1.0 for the other trust relations. The trust relations and their corresponding adjacency matrix are shown in Figures 1A,B, respectively.

By summing up all the values in all the rows, we will get what we can call **D**_**actual**_. If we divide the **D**_**actual**_ value with the **D**_**max**_ value, the sum of all rows if they all were 1.0, then we get the **D** value. In the example from Figure 1, it would be $\frac{D_{actual}\;}{D_{\max}}=$ = 12/16 = 0.75, that is, the density of the group would be 0.75. To illustrate how the density value changes dependent on the relations between the group members, let us change trust relations a little to D**T**A = 0.3, A**T**D = 0.4, D**T**B = 0.3, B**T**D = 0.2, and keep the rest at 1.0 (illustrated in Figures 1C,D). The new group **D**_**actual**_ would be 13.2, which would give us **D** = 13.2/16 = 0.825, which means that the new group has a higher density value than the first group. The density value of a CGA should be understood as:

**D** = 1 =_df_ There is a full connection between the group members.
**D** = 0 =_df_ There is no connection between any group members.
**D** = x, where 0 < x ≤ 1 =_df_ There is a connection between some members in the group.

The implication is that if all trust relations between the group members are 0, then the CGA will cease/dissolve.

### Intensity of cooperative group activity

We have now established a formal way to estimate the compatibility of the member's subplans in a CGA by the **MS** function and the connections between the agents in a shared intention by the **D** function. These two phenomena are not truly distinguished from each other, of course, but separate enough to make it intelligible to analyze them separately. The characteristic of both phenomena is that they are both very important for the survival and duration of a CGA.

One can also look at the product of the compatibility and density, **I** = **MS** × **D**. The value of **I** will determine the intensity of the CGA. The intensity of a CGA, seen in the formal definition, is the degree of the CGA based on the trust relations between the members in the group and the compatibility of subplans that these members have relative to that intention. Following the same formulation as before:

**I** = 1 =_df_ The intensity of the CGA is maximal.
**I** = 0 =_df_ The CGA has no intensity.
**I** = x where 0 < x ≤ 1 =_df_ The CGA has intensity.

If a CGA lacks intensity, the CGA ceases to be. There are two ways in which a CGA can be characterized as having **I** = 0, either by M(α_n_,β_m_) = 0 or by **D** = 0. The formulation of **I** makes it possible to compare two or more groups that have a similar CGA but are constituted of different members, for example, different supporter teams. This will not be pursued here, but instead we will focus on how a CGA's degree of intensity can increase or decrease.

Friedkin et al. showed how an individual's belief about X depends and changes with (i) whether the individual finds the credibility of X dependent on the credibility of other beliefs Y and Z (the logical structure) and (ii) whether or not the people they trust (interpersonal influence) find X, Y, and Z credible (Friedkin et al., 2016). With their model, they illustrate the mechanism of “group thinking” in small groups that even if the members start with divergent initial beliefs and divergent attitudes about the logical structure, the member ends up converging in their beliefs as long as there is one person with interpersonal influence (which would be high trust in this paper's terminology). In a recent paper, Rawlings and Friedkin used the Urban Communes Data Set (Martin et al., 2001) to evaluate the relational tensions in sentimental networks over 2 years. The dataset was divided into high- and low-commitment communes based on the UCDS field setting investigators. Although all communes were formed around a collective identity with the explicit goal of fostering community, the high-commitment groups had the following four features:

“(1) the overall purpose of the commune is transcendent, (2) the legitimation of commune leadership is partly or wholly charismatic, (3) some or many rules exist that govern members' conduct and behavior, and (4) a strong feeling exists among the members that the commune is a “We.” Nine of the 31 communes have all of these features, and we take them to be high-commitment communities” (Rawlings and Friedkin, 2017, p. 526).

Although (2) and (3) can play a nontrivial role in the sustainability of a CGA, it is outside of the scope of the current paper, whereas (1) and (4) are highly relevant. They found that tensions (negative feelings toward each other) among individuals in high-commitment communities decreased more over time than in low-commitment communities. Additionally, high-commitment communities were more characterized by an elevated, friendly sentiment structure (a friend of a friend is a friend, an enemy of a friend is an enemy) compared with low-commitment communities that elevated agonistic sentiments structures more (a friend of an enemy is an enemy).

Based on the above, we can crudely postulate that with every shared intention, there is an inherent mechanism to increase the value of **I**. This inherent function cannot be explained by the shared intention alone, which would mean that, if true, it is a consequence of our tendency to avoid cognitive inconsistency and some byproducts of our need to “tune in” to each other and other social needs. I will not discuss this further and will just presume that such a mechanism exists.

We can call the mechanism the increment of **I** and formally denote it as INC(**I**), where INC(**I**) is the function that takes **I** = 0.5 at time t_1_ to **I** = 0.75 at time t_2_. That the INC(**I**) increases could either mean that the **MS** value increased, the **D** value increased, or both. Although simple, this model of the mechanism behind the sustainability of a CGA can explain and explore how and why cognitive change happens when engaging in a CGA.

### Examples

To illustrate some properties of INC(**I**), we will apply it to several social situations.

#### Regular friendship

An important part of friendship is participating in different CGA, not the least the CGA of being friends. Let us say that Arthur and Bruce want to be friends, but they have very different beliefs about most things or that their **MS**(α,β) is very low. Since INC(**I**) and D = 1, their **MS** must increase. This can be done in several ways. One way is that one of them explicitly pressures the other to change their beliefs. Another way is that Arthur and Bruce slowly stop involving their preference in their beliefs and plans to the extent that fewer subplans are important for them concerning the shared intention. This can be stated as that *A* ∩ (*X*|φ_*t*1_) = {α_1_, α_2_…α_*n*_} evolves to *A*∩(*X*|φ_*t*2_) = {α_1_, α_2_…α_*k*<*n*_}, meaning that ϕ at time t_1_ when the shared intention X (playing together) was possible is different from φ at time t_2_, to the extent that the set of subplans is important for Arthur relative to the shared intention is smaller than before. This reflects the fact that, in the end, some people just want to get along.

#### Peer pressure

Suppose that Arthur, Bruce, and Clark are friends and that Bruce and Clark want Arthur to steal some apples, something that goes against Arthur's preference, that is, something Arthur has no plan to do. We can characterize this situation as their **MS** = 0. However, this would mean that the shared intention between Arthur, Bruce, and Clark of playing that day or even being friends will stop, something that we can presuppose Arthur does not want. To obtain **MS** > 0, Arthur must change some of his subplans, or Bruce and Clark will have to. Given INC(**I**), the more efficient (and from this framework more plausible) alternative is for Arthur to change or drop some subplans.

#### New friends

Following the example in Density as group complexity, we envision Arthur, Bruce, and Clark as friends that meet every Tuesday for an after-school jigsaw activity, and Diana, a neighbor to Clark, tags along on Tuesday. Let us presuppose that, given this situation, they have **MS** = 1, and we know that they have **D** = 0.75 (as illustrated in Figures 1A,B). When Bruce and Clark go to the bathroom at time t_1_, it is more or less just the case that Arthur and Diana happen to be in the vicinity of each other doing a jigsaw puzzle, which means that their subgroup **I** would be of very low value, if existing at all. However, given INC(**I**) at the end of the after-school jigsaw activity at time t_n_ when Bruce and Clark leave again, it is not the case that Arthur and Diana just happen to be in the vicinity of each other they are doing a jigsaw puzzle together. What has happened is that given INC(**I**) and the shared intention that **I** is based upon, Arthur's and Diana's trust relations have increased and they have become more connected (as illustrated in The common knowledge problem and Figures 1C,D).

#### Dividing into subgroups

Imagine a group composed of A, B, C, D, E, and F with a low **I** based on a low **D** value. The problem is not that their subplans and beliefs do not mesh, and because of INC(**I**) the group probably splits into two groups A, B, C and D, E, F where both groups have **I** = 1 or close to it.

#### Bringing in a generalized other from a larger group

One difference between shared and collective intention is that there is a generalized other in the latter. This means that the members do not need to know about each other, only that there are other members. This will change how the **D** looks and works and, in extension, how the INC(**I**) will work. For the present purpose, it suffices to acknowledge that in a collective intention, there is no efficient way to change these generalized others' subplans, so given INC(**I**), if a member, Arthur, wants to continue to be part of the CGA, then Arthur, probably drop/change his subplans.

This phenomenon is not unique, for example, consider Arthur, who wants to join a church. This church has a collective belief with many subplans and individuals involved. Arthur's belief before participating in the collective belief of the church was that he believed that there is a God. Given this situation, the rest of Arthur's subplans are compatible with all the other beliefs of the collective belief of the church but are not fully compatible. Now, given INC(**I**), what will happen is probably that the **D** value will increase, that is, Arthur will get to know the others in the church. Since all these new relationships are based on the same collective intention in the same situation, that is, the church, it means that given the INC(**I**) of all the subgroups, Arthur will not only drop but also actively change some of his subplans. One can think that when Arthur first entered the collective belief of the church, he did not believe that God was the father, the son, and the holy spirit, but he thought it is possible to the extent that they had a very low **MS** value. Nevertheless, after a period, Arthur would have changed his subplans to the extent that his subplans are fully compatible with the belief that God is the Trinity.

That Arthur's subplans became more compatible with the collective intention of the church was, of course, the whole point of Arthur's engagement with the church in the first place. I am not claiming that there was not much thinking on Arthur's part and many active choices. What this example illustrates is that from the framework of INC(**I**), we can isolate and explain some part of how beliefs spread between members of a group and focus on some of the mechanisms that explain the gradual and unnoticed changes in an individual's beliefs after engaging in a new collective intention.

To make it easier to quantify over a set of subplans, we separated the emotional level of a belief from the subplans. However, the emotional attitudes on the subplans are very important when analyzing the INC(**I**) in many situations. We need to go back to the definition *A* ∩(*X*|φ) = α to see how this is the case. This formula tells us that, in general, only the subplans relative to the situation given the shared intention are relevant in analyzing a shared intention. However, it is not an uncommon phenomenon that when an individual has started to engage in a CGA (be it a new friend group, religious, ideological, political activity, or when they are in love, etc.) that the beliefs associated with those activities influence the activity in other areas.

#### Effects on friendship

We think of two friends, namely, Arthur and Bruce. They like to talk about anything. Now, say that Arthur joins the church as mentioned above, and after a while, Arthur becomes very religious. Since Arthur is now very religious, he will probably invest cognitively and emotionally into religious matters. Before when Arthur and Bruce met, they sometimes had discussions about X and the subplans relevant to the CGA that their discussions were {α_1_, α_2_… α_n_} and {β_1_, β_2_… β_n_}, and they were compatible. But, after Arthur has become religious, there are some subplans added {α_1_, α_2_… α_n_ ∪ α_R1_, α_R2_… α_Rn_}, which is not compatible with β^4^. What has happened here is that both sets of subplans of *A* ∩ (*X*|φ) = α and *B* ∩(*X*|φ) = β have changed. In the case of Arthur, it is his overall set of beliefs that has changed, that is, the left side of the intersection has changed, whereas in the case of Bruce, it is the right side of the intersection that has changed, that is, it cannot be the same situation (φ) or intention (X) as before. Thus, the addition of ideology beliefs (involving both fact and preference types, beliefs, which in turn affect the logical structure of beliefs) to Arthur's set of beliefs will change the circumstances of the context (φ) that individuals such as Bruce have to be in if he aims at a CGA with Arthur.

The result is that the relationship between Arthur and Bruce will either have some religious aspect to it, or they will need to be involved in a shared intention that Arthur's religious beliefs cannot spill over into, like only discussing soccer. Given INC(**I**), there are a few possible scenarios:

(a) The **MS** value increases to the effect that Bruce becomes more interested in the religious stuff at the same time as Arthur changes more of his beliefs and subplans.
(b) The **MS** value increases to the effect that the things that Arthur and Bruce engage in together are either related to religious matters but not too much or things that are completely different, e.g., soccer. More explicitly, the **MS** value increases because the number of activities they can do together decreases.
(c) Because of (b) and not (a) the decreases in activities and the effort and resources it takes for Bruce to maintain or increase the **I** value, given INC(**I**), the relationship between Arthur and Bruce eventually fades out.
(d) The MS value increases to the effect that Arthur becomes less religious.

From the framework of INC(**I**), there are some interesting analyses of this. First, even if (d) is possible, it is the least plausible. I do not mean to say that religious and political people do not want to keep old friendships, only that it is cognitively costly to moderate the beliefs one is emotionally involved with and highly prefers. From the point of view of INC(**I**), it will be a question about what is most efficient, which could result in the break of the CGA or the friendship. Second, scenario a) illustrates one way of making a person change their belief is to put the relationship at stake. This does not have to be explicit; it could just be the more discreet change that follows from the INC(**I**) that in order to keep a steady **I**, the other needs to change their beliefs and subplans. One way this cognitive change can happen is for Bruce to deflate or inflate the meaning of a proposition. Given the consistency theory, individuals try to resolve the tensions between two propositions (X and Y that they hold) if they believe that not X follows from Y (Gawronski and Brannon, 2019). Let X be “Arthur is reasonable” and Y be “all religious individuals are unreasonable”, then there is an inconsistency between X and Y. By deflating the scope of Y to be “many/some/not all religious individuals are unreasonable”, then X and Y are consistent. Let Z be the proposition “Arthur's religiosity is based on a revelation” and Y be “it is only reasonable to change one's belief based on scientific literature and empirically verified facts”, then not X would follow from Y and Z. However, by inflating Y to be “it is only reasonable to change one's belief based scientific literature and empirically verified facts *and revelation*”, then the propositions X, Y, and Z are consistent.

Third, even if there is a mechanism, that is, the INC(**I**), that pushes b) to become c), some individuals put effort into the relationship to keep it alive. The INC(**I**) framework highlights that Bruce will have to do much heavy lifting to maintain the relationship with Arthur without falling into (a). Moreover, if (a) happens, there has been a substantial cognitive change for Bruce, that is, a change in his beliefs, attitudes, and preferences as a result of the sustainability of the CGA.

## Empirical outlook

The aim has been to analyze concepts and constructs involved in shared intentionality to the effect that this framework will help explain and explore the relationship between cooperative group activity, cognition, and development. In doing so, several empirical postulations have been made where the two more central ones are: (i) creating and sustaining a CGA have different cognitive requirements and (ii) engagement in a CGA stimulates cognitive change in its members. In this section I present additional empirical support for them and derive empirical hypotheses.

### Creating and sustaining a CGA have different cognitive requirements

A contrary postulate to explain the duration of a CGA would be that we continuously recreate the CGA or when there is a change in the dynamic (e.g., a subplan is changed) a new CGA must be created. These contrary alternatives face trouble explaining the continuations of cooperative behavior such as 3-year-olds distribute the spoils of a CGA fairly, or close to fairly, among its members but not spoils after noncooperative activity or to others not part of the CGA (Hamann et al., 2011; Warneken et al., 2011), or continue to collaborate even if they can get the reward by themselves (Rekers et al., 2011). These findings are expected in the current account since the shared intention and its subplans are still at play, since the CGA is sustained until it is terminated. Given that there is a difference between creating and sustaining a CGA (as argued in section Consequence of creating a cooperative group activity and Sustainability of cooperative group activity), the question is whether they have different cognitive requirements. Since the sustaining of a CGA will involve changing a subplan in order to reach consistency and meshing, it requires both metacommunication (de Haan et al., 2021) and some form of cognitive flexibility of the individual. This would mean that a sufficient level of cognitive flexibility and executive function is required. A conjecture would be that in early childhood, the ability to sustain a CGA (measured in duration and change to plans) would be positively associated with the level of cognitive flexibility and executive function.

### Engagement in a CGA stimulates cognitive change in its members

A paradigmatic example of a group activity that separates a CGA from mere coordinated group activity is pretend play among peers (Rakoczy, 2006). Several studies have investigated the relationship between social pretend play and cognitive abilities. In a study of 3-year-olds, a strong association was reported between the engagement of pretend play during free time and cognitive self-regulation (Slot et al., 2017). In a recent study of 3.5–5-year-old during 15 h of free play, it was found that the frequency of social pretend play, but not social nonpretend play or solitary pretend play, predicted executive function half a year later (White et al., 2021). Although solitary pretend play has been related to cognitive abilities (Carlson et al., 2014) and social nonpretend play, such as playing a game can be a CGA it can also be done as a mere coordinated group activity, it is, according to this account, the sustaining of the pretend play (i.e., the CGA) that will stimulate additional cognitive change/development. Given this framework and previous studies, a conjecture would be that social pretend play with peers based on minimally cooperative groups would increase executive functions more than social nonpretend play or solitary pretend play, in preschool children. In evaluating these two conjectures, the framework of analyzing the opening, the main body, and the closing of a CGA would be fruitful (Heesen et al., 2017).

## Conclusion

The account presented here is a cognitively plausible description of shared intentionality and a CGA that illuminates the different criteria and cognitive mechanisms involved in creating and sustaining a CGA. By separating the creation and continuation of a CGA, new research questions arise about cognitive change and cognitive development. Social interaction stimulating cognitive development is an old idea in developmental and social psychology, prominently associated with psychologists such as Lev Vygotsky. A framework, such as the one presented here, contributes to a way of exploring different types of group activities (e.g., cooperative vs. coordinated) and their effect on cognitive development from an interdisciplinary approach of psychology, philosophy, and network science. The formal framework of belief compatibility, group density, and intensity was developed to study, compare, and model different groups with the same shared intention but constitutive of different members. Why different groups, and types of groups, affect their members differently are important questions that needs to be confronted for us to understand our cognitive development.

## Data availability statement

The original contributions presented in the study are included in the article/supplementary material, further inquiries can be directed to the corresponding author/s.

## Author contributions

The author confirms being the sole contributor of this work and has approved it for publication.

## Footnotes

1 I defend a version of the truncated approach to the common knowledge problem in Skau (2015).

2 “Derivation: An entity A is derived from another entity (or manifold of entities) B just in case B is required for bringing A into existence (but not necessarily thereafter, for maintaining A in existence) [...] Contingency: An entity A is contingent upon a separate entity (or manifold of entities) B if and only if B is required for A's continued existence (maintenance)” (Thomasson, 1996, p. 293).

3 This is one of many ways one can extract the M and MS values. That one must appeal to the principle of indifference as I have done here is just for connivance and not a necessity.

4 R1 to Rn in {α_R1_, α_R2_… α_Rn_} stands for different religious belief.
